# Looking inside the Ocean: Toward an Autonomous Imaging System for Monitoring Gelatinous Zooplankton

**DOI:** 10.3390/s16122124

**Published:** 2016-12-14

**Authors:** Lorenzo Corgnati, Simone Marini, Luca Mazzei, Ennio Ottaviani, Stefano Aliani, Alessandra Conversi, Annalisa Griffa

**Affiliations:** 1 Institute of Marine Sciences (ISMAR) in La Spezia, National Research Council of Italy (CNR), Forte Santa Teresa, 19032 Pozzuolo di Lerici (SP), Italy; simone.marini@sp.ismar.cnr.it (S.M.); luca.mazzei@sp.ismar.cnr.it (L.M.); stefano.aliani@sp.ismar.cnr.it (S.A.); a.conversi@ismar.cnr.it (A.C.); annalisa.griffa@sp.ismar.cnr.it (A.G.); 2 OnAir srl, Via Carlo Barabino 26/4B, 16129 Genova, Italy; ennio.ottaviani@onairweb.com

**Keywords:** content-based image recognition, feature selection, gelatinous zooplankton, autonomous underwater imaging, GUARD1

## Abstract

Marine plankton abundance and dynamics in the open and interior ocean is still an unknown field. The knowledge of gelatinous zooplankton distribution is especially challenging, because this type of plankton has a very fragile structure and cannot be directly sampled using traditional net based techniques. To overcome this shortcoming, Computer Vision techniques can be successfully used for the automatic monitoring of this group.This paper presents the GUARD1 imaging system, a low-cost stand-alone instrument for underwater image acquisition and recognition of gelatinous zooplankton, and discusses the performance of three different methodologies, Tikhonov Regularization, Support Vector Machines and Genetic Programming, that have been compared in order to select the one to be run onboard the system for the automatic recognition of gelatinous zooplankton. The performance comparison results highlight the high accuracy of the three methods in gelatinous zooplankton identification, showing their good capability in robustly selecting relevant features. In particular, Genetic Programming technique achieves the same performances of the other two methods by using a smaller set of features, thus being the most efficient in avoiding computationally consuming preprocessing stages, that is a crucial requirement for running on an autonomous imaging system designed for long lasting deployments, like the GUARD1. The Genetic Programming algorithm has been installed onboard the system, that has been operationally tested in a two-months survey in the Ligurian Sea, providing satisfactory results in terms of monitoring and recognition performances.

## 1. Introduction

Automatic recognition of plankton is a rapid expanding field [1] and many approaches exist for the recognition and classification of micro-zooplankton specimens [2,3]. Nevertheless, very few literature refers to the detection and classification of macro gelatinous zooplankton.

The dynamics of marine plankton populations are still largely unknown, especially in the open and interior ocean, even if the number of observations collected in the last decades is growing. Marine zooplankton plays a key role in the trophic chain, due to the fact that most marine animals have life cycles including a planktonic phase and very often feed on zooplankton for their entire life or part of it [4].

Gelatinous zooplankton is a crucial actor in the trophic mesopelagic communities, with implications for the carbon cycle [5] and for fisheries, since it often competes with fish for food sources, and often is a critical indicator and driver of ecosystem performance and change [6].

Mounting evidence suggests a rapid change in the structure of pelagic ecosystems from one dominated by fish to a less desirable gelatinous state, bearing critical ecological, economic and social consequences [7]. Several studies indicate that such alterations are happening in different ecosystems [6,8,9,10,11,12]. In general, eutrophication, human-induced stresses, climate change, translocation and habitat modification appear to be promoting jelly blooms to the detriment of other marine organisms [7,13].

Invasions of gelatinous zooplankton, jellies hereafter, have been reported as possible causes of major ecosystem changes, or regime shifts, and, in addition, some cnidarians may pose risk for tourism because the medusae stings are considered highly offensive to summer bathers [11]. Such concerns have promoted the start of several programs, such as the Catalan “Medusa Project” [14], the CIESM “JellyWatch Programme” [15], and the “Spot the Jellyfish” initiative [16], aimed at monitoring jellyfishes and at giving early warning against their invasions.

Mapping of gelatinous zooplankton (e.g., pelagic cnidarian and ctenophore) is therefore necessary to understand their biogeographical distribution, to monitor changes in their abundance and to early detect invasions. Classical sampling with towed plankton nets is not appropriate for these delicate organisms, and furthermore is usually expensive. To overcome these shortcomings, imaging techniques can be used for the automatic monitoring of this group.

In general the automatic recognition of jellies deals with several issues having different nature, ranging from physics and biology to technology. The large number of jelly species, the non-rigid shape, the almost transparent body, the size of organisms and their capabilities to be almost invisible to possible predators make it difficult to identify an effective approach for the automatic recognition of these organisms.

Moreover, the particular acquisition context, i.e., light conditions, water turbidity, deformation typical of the underwater images, light reflection close to the surface, lack of natural light in deep water, presence of non-relevant floating objects (e.g., fishes, litter, algae, mucilage), strongly affect the image quality and thus the recognition performance.

On the technological side, the sensitivity of the imaging instrument, the sensor noise, the selected field of view and the lighting system capabilities play a crucial role in the recognition quality.

Finally, the pattern recognition methods have huge influence on the detection performances, which are highly related to the image pre-processing steps (e.g., contrast, color and contour enhancement), to the identification of the regions of interest, to the extraction of image features and to the selection of relevant image features.

Many different imaging systems have been developed in the last years for the automatic monitoring and investigation of gelatinous zooplankton, but no one combines the capability of being hosted on both fixed platforms (e.g., oceanographic moorings and submerged observatories [17,18]) and mobile platforms (e.g., Autonomous Underwater Vehicles (AUV) [19], sea gliders [20], drifter buoys [21] and ARGO float (http://doi.org/10.17882/42182) vertical profilers [22]), with performing onboard automatic recognition of jelly specimens over a large dimension range. Table 1 presents the main zooplankton imaging devices comparing the core features of each system.

This paper presents the GUARD1 imaging system (EU patent application EP14188810) designed and developed for autonomous, long term and low-cost monitoring and recognition of gelatinous macro-zooplankton in order to assess their abundance and distribution. Moreover, the performance of three methodologies for the automatic recognition of jellies are discussed and compared in order to select the one to be operationally run onboard the system.

GUARD1 is the improvement of the proof of concept discussed in references [36,37,38], based on more powerful software control components and cheaper electronics, in order to optimize the acquisition strategy and have costs compatible with disposable instrumentation. It is a low-power and stand-alone system conceived for installation on both fixed and mobile platforms for acquiring images of objects or organisms from 1 mm to 100 cm in size. Onboard the device, the image content is analyzed, recognized and classified, with the aim of monitoring the ocean interior at various scales in space and time. The system is completely autonomous, uncabled and designed for long lasting deployments. These characteristics make the GUARD1 complementary to the devices discussed in Table 1.

The pattern recognition approaches investigated hereby are binary image classifiers based on Support Vector Machines (SVM) [39], on Elastic Net based on the Tikhonov regularization (TR) [40] and on Genetic Programming (GP) [41]. These three methods are well established in literature, but presently TR and GP are not commonly used for underwater image classification tasks, even if recently GP has been applied to automatic detection of manganese nodules in sea floor images [42].

The proposed methodologies have been integrated with feature selection schemes for identifying the most suitable image features capable to discriminate jellies from other floating objects captured by the images (e.g., fishes, litter, algae, light reflections), in order to optimize the recognition performances and the computational cost. The latter is a key requirement for algorithms to be run onboard an autonomous system designed for long lasting deployments, since it affects the system energy consumption.

This paper is organized as follows: Section 2 presents the developed imaging system, describes the image acquisition strategy, the image processing workflow, the pattern recognition and supervised learning framework and the three experimented methodologies. Section 3 reports the achieved results and Section 4 analyzes them in terms of recognition performances and feature selection stability. Section 4 also describes the operational test performed on the system running the selected recognition method. In Section 5 the overall system functionalities and performances are analyzed and conclusions are given.

## 2. Experimental Section

### 2.1. GUARD1 Imaging System

**Figure 1:**
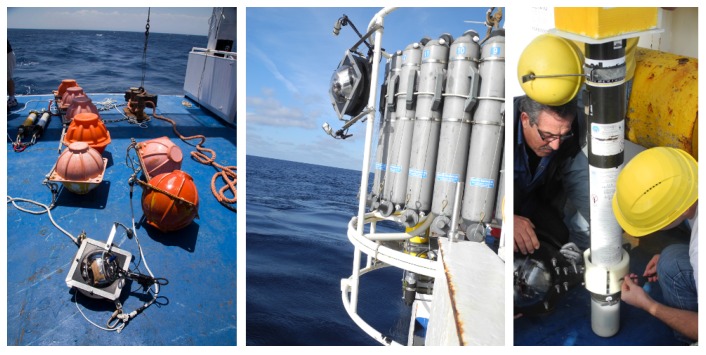
The GUARD1 autonomous imaging system in three of its possible configurations: on the left panel it is hosted on a mooring chain; in the middle panel it is mounted on a rosette; on the right panel it is hosted onboard an ARVOR profiler.

The system includes a low power and low cost image acquisition device to collect time series, coupled with on board hardware and software modules for managing the image acquisition, analysis, recognition and content communication as discussed in reference [38]. It was tested on fixed (coastal station and moored) and mobile platforms (drifter buoys and Argo floats) and mounted onboard an oceanographic rosette operated by a research vessel. Figure 1 shows the instrument in different scenarios.

The instrument is fully programmable for operating in a wide range of applications and it is built up of three modules: (i) the acquisition component; (ii) the elaboration and storage module and (iii) the control module.

The acquisition component (i) consists of a programmable consumer camera, housed for underwater operations. It is equipped with a lighting system that is automatically turned on, through the use of a light sensor, only if the natural light is not sufficient for the specific acquisition purposes.

The sampling strategy and the programming of the acquisition parameters (e.g., ISO, exposure time, focal length, iris aperture) are designed for the automatic adaptation to the lighting conditions thanks to a specific modification of the firmware controlling the camera. In particular, with respect to the system release described in [38], the LUA script based on CHDK [43] running on the camera firmware has been improved by implementing a more layered analysis of the camera light sensor response. As a result, the acquisitions are triggered reducing the lighting system usage, thus reducing the system power consumption.

The elaboration and storage module (ii) consists of a CPU board hosting and running algorithms for image analysis and pattern recognition. These algorithms are responsible for the different tasks of the image interpretation pipeline. The analysis algorithm extracts the relevant features from the acquired images, while the pattern recognition algorithm uses the extracted features to recognize the relevant image content (e.g., number of specimens, relevant image sub-regions) [44,45]. The recognized image content can be stored and/or communicated outside the instrument. In order to save up the battery pack, the image elaboration algorithms works on groups of images (not on single images) as they run at scheduled time intervals.

The communication module is now in the process of being developed. A high-level design has been realized and the communication architecture and the devices to be employed have been identified. In particular, the communication block will operate a dual configuration, based on acoustic and electromagnetic channels.

The acoustic channel will be used for communicating with submersed fixed or mobile platforms in the vicinity of the system. The GUARD1 will host an acoustic modem, acting as the master in a master-slave configuration, periodically pinging the slave modems eventually located in the surroundings. The slave acoustic modems would be installed onboard submarine mobile platforms (e.g., AUV) patrolling the area or that are in the vicinity on their mission track, or onboard fixed platforms (e.g., fixed submersed observatory) the GUARD1 might approach during its mission. At the moment the software modules for the acoustic communication pipeline have been implemented and are in their test phase, while the performances of different low-cost and small-size acoustic modems are now being analyzed and compared.

On the other hand, a sea-surface proximity sensor will be mounted onboard the GUARD1, in order to trigger the electromagnetic communication in case the system is surfacing. This communication will be performed using GPRS (for coastal activities) or satellite modems equipped with adequate antennas, and will transfer data to a central station. The first tests for this configuration will begin in the next few months.

Since the communication module is now under investigation and it has not yet been implemented in an operational release, at the moment the information extracted by the elaboration component can be accessed after the instrument recovery.

The control module (iii), responsible for the management of the operational workflows of acquisition, image elaboration, communication and lighting modules, has been redesigned with respect to the system release presented in [38]. The new release is based on cheaper electronic components, thus reducing the system costs and making them compatible with disposable instrumentation ones. The control block allows for the acquisition frequency programming and the start-stop scheduling of the elaboration component. The control component tasks can be programmed through a remote controller.

### 2.2. The Survey

In the period May–June 2013, a survey has been carried out in Baia Santa Teresa, in the Ligurian Sea close to La Spezia. The monitored area is a small and closed bay adjacent to the Gulf of La Spezia.

The GUARD1 system was operated on a fixed platform in the center of the bay at a depth of 5 m. Images were captured every 5 min with a resolution of 640 × 480 pixels and a color depth equal to 8 bpp.

9282 images were acquired and then converted to gray scale. From the collected images, 14,522 Regions of Interest (RoI) were automatically extracted, based on the approach described in Section 2.3, to be processed by the pattern recognition algorithms.

Among the identified 14,522 RoIs, 545 have been annotated by a team of marine biologists and experts of gelatinous zooplankton, in order to identify gelatinous macro-zooplankton specimens, suspended particulate, light reflections, fishes and floating objects like algae and litter. This dataset was used for the training and validation of the three recognition methods.

In a second stage, after having performed the comparison among the three recognition methods, all the remaining images have been annotated by the team of marine biologists and experts of gelatinous zooplankton, in order to build the ground-truth dataset to be used for the evaluation of the performances of the recognition method selected to operate onboard the GUARD1 system. The results of this evaluation are presented in Section 4.

### 2.3. Image Processing and Feature Extraction

The core characteristic of the GUARD1 system is the capability of automatically extracting and identifying information from the acquired images. The achievement of this task is possible thanks to the elaboration component that hosts and runs algorithms for image processing and content recognition.

The aim of the image processing task is the extraction of the image-features to be used for the recognition of the relevant objects (jellies in this case) to be identified. An image-feature captures a descriptive characteristics of an image: in general it is based on the analysis of colour, texture, shape and salient points [45]. It can be defined as global when it refers to the whole image or as local when it refers to a subset of pixels.

The scientific literature on Computer Vision is rich of approaches for extracting and combining features in the context of image recognition, classification and retrieval [44,45,46,47], also in marine science applications, where several techniques have been investigated for the extraction of even complex features [3,25,48].

Since the GUARD1 system is a stand-alone device designed for long lasting deployments, it has a limited computational capabilities of the elaboration component due to the essential requirement of limiting the power consumption as much as possible. Thus, the algorithms developed for the feature extraction have been designed to identify the simplest (i.e., requiring the lowest computational load) possible features in the collected images. As shown in Section 3, an appropriate use of these simple image-features provides good results.

The proposed image processing workflow reflects a typical bottom-up image processing sequence of operations, whose steps are briefly outlined in the following paragraphs, in the order of their application.

#### 2.3.1. Image Enhancement

**Figure 2:**
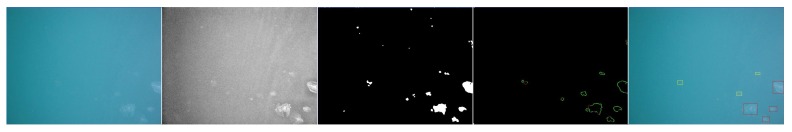
An example of the image processing steps. From left to right: original image, CLAHE enhancement of the original image, background/foreground segmentation, contours extracted from the original image by the Sobel operator, Region of Interest (RoI) identification.

The Contrast Limited Adaptive Histogram Equalization (CLAHE) algorithm [49,50] is used to improve the foreground/background contrast of potentially interesting image subjects. CLAHE calculates histograms of small adjacent regions of pixels that are equalized separately. The overall equalized image is obtained as a bilinear combination of the equalized neighbour regions. Dealing with images of the water column (i.e., uniform and low-textured background), CLAHE has proved to provide a very natural enhancement, avoiding artifacts due to excessive gain in low-textured areas, as shown in Figure 2. This step has a low computational impact in the image preprocessing activities since it is linear in the number of image pixels.

#### 2.3.2. Background-Foreground Segmentation

Like in many computer vision applications, the foreground must be segmented from the background, in order to detect objects inside the images. In this work it is supposed that the background can be modelled as a slowly varying 2D function. Many approaches are available for defining segmenting filters based on this assumption, either linear (moving average) and non linear (morphological) [51]. The GUARD1 system uses a simple box-shaped moving average filter [52], with a box area of a size comparable with the size of expected objects (order of 20 pixels). This filter transforms the original image into a binary image that discriminates the image foreground from the image background. After this step, the foreground image regions (blobs) can be defined as the set of pixels with intensity value higher than the background and exceeding a given global threshold. This filter has been efficiently implemented linearly in the number of pixels through the use of the integral image approach [53]. The information about the radiometric nature of the jellies, that appear brighter than the surround when illuminated, is used to tune the background/foreground segmenting filter.

#### 2.3.3. Region of Interest (RoI) Identification

This step operates on the blobs obtained by the background/foreground segmentation and identifies all the connected foreground regions that are candidate to be interesting objects (RoIs). Dealing with underwater images, blobs representing light reflections or suspended particulate should be avoided as soon as possible. Differently from the relevant subjects (i.e., jelly specimens), these kind of blobs are characterized by a blurred contour that can be easily identified by analysing the internal/external contour gradient. The gradient analysis is performed by a filtering process based on the Sobel operator [54] applied to the original image (i.e., not binarized). The contours obtained by the Sobel operator are then compared with the blob contours extracted by the binary image. If the number of pixels of the Sobel contour is less then the 50% of the pixels’ number of the morphological contour obtained by the binary image, then the blob region is not considered relevant. In fact blurred parts of the subject contour are not identified by the Sobel operator implemented with a small size kernel (e.g., 3×3); on the contrary, foreground subjects are characterized by contour pixels that have a strong gradient (i.e., identified by the Sobel operator) and an evident morphological characterization (i.e., binary image). The computational cost of running the Sobel operator is linear in the number of image pixels.

#### 2.3.4. Feature Extraction

Finally, the validated RoIs must be processed in order to extract a feature vector to be used in the recognition task discussed in Section 2.4. The extracted features belong to two groups: the geometric one, based on the shape of the RoI, and the texture one, based on the distribution of the grey levels inside (and outside) the RoI, as summarized in Table 2.

The length of the minor semi-axis (*semiAxm*) and the length of the minor (*axm*) and major (*axM*) axes of the RoI oriented bounding box describe the size of the relevant subject.

The eccentricity (*ecc*) describes how much the RoI differs from a circle and it is defined as the ratio of the foci distance and the major axis of the RoI surrounding ellipse. The eccentricity varies between 0 (i.e., no eccentricity, the RoI is a circle) and 1 (i.e., maximum eccentricity, the RoI is a straight line).

The solidity (*solidity*) is the ratio between the area of the RoI and the area of the corresponding convex hull. The more the solidity tends to 0, the more the RoI border is jagged; on the contrary, the more the solidity tends to 1, the more the RoI border is smooth.

The area and the perimeter of the RoI (*areap*, *perimeter*) correspond to the number of pixels contained inside and on the border of the RoI, respectively.

The histogram shape index (*histIndex*) captures the overall pixel intensity variance inside the RoI. It is obtained by transforming the RoI into a grey level image and extracting the histogram *h* of the pixel intensities. Then the *histIndex* is defined as the standard deviation of *h* after the histogram normalization $\left(\frac{h}{\sum h}\right)$.

Similarly the standard deviation of the mean grey level (*stdg*) captures the variation of the pixel intensity with respect to the RoI mean grey *μ*.

The entropy (*ent*) of *h* captures the information stored in the RoI, and finally, the normalized contrast index (*contrast*) is defined as the ratio between the difference in the mean grey level inside the RoI (mean(gInt)) and outside the RoI but within the oriented bounding box (mean(gExt)), and the mean grey level inside the whole bounding box.

All the previously discussed image features were chosen such that they have a linear computational cost with respect to the number of pixels. Moreover, though a single image feature can appear not relevant with respect to the objective of jelly recognition, it can become relevant if combined with other image features. This point is crucial and it is discussed in the following sections within the presentation of the methodologies for the jelly recognition and the discussion of the obtained results.

Figure 2 shows an example of the image processing steps according to the proposed workflow.

### 2.4. Image Recognition

The recognition problem faced in this work corresponds to the detection of one or more jelly specimens within the analysed images. To achieve this task, a binary classifier is defined on the RoIs identified in the input images, where the returned output assumes the value 1 if the RoI, as defined in Section 2.3.4, contains a jelly specimen, and 0 if the RoI does not contain any jelly specimen [37]. Information on the specimen location inside the analysed image is provided by the coordinates of the calssified RoI within the image.

Three methodologies for defining the binary classifiers have been experimented in this work: Elastic Net based on Tikhonov regularization (TR) [40], Support Vector Machines (SVM) [39], and Genetic Programming (GP) [41]. Particular focus has been put on the capability of each method in selecting the most suitable image features able to discriminate gelatinous zooplankton from other floating objects present in the images. The binary classifiers are built with a supervised machine learning approach [55]: an annotated dataset of images captured by the GUARD1 device, i.e., a representative set of positive and negative examples (as described in Section 2.2), is the ground-truth used for training and validating the classifiers. The ground-truth nested within a K-fold Cross-Validation (CV) framework [56,57] provides a quantitative estimate of the recognition performance. In Section 3, the more effective notion of accuracy will be defined and used in place of the validation error.

In order to build the positive and negative examples, from each image of the dataset, the RoIs and the corresponding image-features are extracted according to the image processing task described in Section 2.3. In particular, let *n* be the number of RoIs $x_{i}$ extracted from all the images of the dataset, let *p* be the number of features extracted from each RoI and let $y_{i}\in \left\{1,0\right\}$ be the label of each RoI. The set of pairs

(1) $$E=\{\left(x_{i},y_{i}\right)\;|\;x_{i}\in X\subseteq R^{p},i\in \left[1,n\right],y_{i}\in Y=\left\{1,0\right\}\}$$

represents the dataset of the image-features used for the training and validation of the binary classifiers.

In the following of this section, the three methodologies used to build the binary classifiers are briefly presented.

#### 2.4.1. Elastic Net Based on Tikhonov Regularization

The method proposed hereby is built up on the elastic net based on Tikhonov Regularization (TR) approach as it has been formulated by De Mol et al. [40]. This approach has been introduced in genomics framework, in order to produce gene signatures capable to address prediction problems from high-throughput data, like in the case of DNA microarray data. The purpose of this method is to reduce the number of features used in the classification to solely the relevant ones. TR is a multivariate analysis approach for pattern recognition embedding a feature selection scheme taking into account the correlation patterns linking the features in cooperating groups. As shown in reference [58], TR is able to select predictive models characterized by both sparsity and low bias, generating stable classifiers even in presence of low cardinality datasets, and it selects models which are asymptotically equivalent in terms of prediction accuracy.

Since it is formulated as a convex problem, this method has a solid mathematical foundation and it can be implemented through simple algorithms requiring low computational resources.

According to the binary classification problem formulation and with reference to the set *E* defined in Equation (1), the relation between $x_{i}$ and $y_{i}$ is modeled as $y_{i}=\beta \cdot x_{i}$, where the attention is restricted to linear functions, that means to vectors $\beta \in R^{p}$. It is also assumed that the input/output pairs $\left(x_{i},y_{i}\right)$ are independent and identically distributed with a fixed but unknown probability density p(x,y) with (x,y)∈X·Y. Under these assumptions, the core of the method is the minimization of the objective function defined by Zhou and Hastie [59]:

(2) $$\frac{1}{n}{∥Y-X\beta ∥}_{2}^{2}+{\mu ∥\beta ∥}_{2}^{2}+\tau {∥\beta ∥}_{1}$$

where *X* is the n×p matrix such that the entry $\left[X_{ij}\right]$ is the *j*-th component $x_{i,j}$ of the image feature vector $x_{i}$ that belongs to the training set *E*, *Y* is the n×1 label vector with $\left[Y_{i}\right]=y_{i}$ and $\beta \in R^{p}$ is the model classifier that maps the image feature vectors $x_{i}$ into the corresponding label $y_{i}$. In this formulation it is assumed that data are normalized to zero mean.

The first term in Equation (2) expresses the approximation error of the classifier *β* with respect to the training set *E*. The second term in Equation (2) enforces the stability and the uniqueness of the minimizing solution by penalizing the $l^{2}$-norm. The third term reduces the number of image features involved by the classifier by penalizing the $l^{1}$-norm of the model vector *β*. The non-negative parameters *μ* and *τ* are called respectively the regularization parameter and the sparsity parameter.

The TR methodology minimizes Equation (2) through a two stage strategy based on an internal CV framework run on the training set *E*: in the first stage (I) TR learns a minimal set of image-features best suited for accurately recognizing the jelly specimens. This is obtained by selecting a small value of *μ* (i.e., applying a LASSO regression [60]) and by varying *τ* within the internal CV framework. The outputs of this stage are: the parameter $\tilde{\tau }$ corresponding to a minimal number of image-features, a new input matrix $\tilde{X}$ and a new model vector $\tilde{\beta }$, both restricted to the selected image-features.

The second stage (II) consists of a regularized least square procedure [61,62], where *μ* increases within the internal CV framework, in order to regularize as much as possible the classifier based on $\tilde{\tau }$, $\tilde{X}$ and $\tilde{\beta }$. Output of the second stage is the value $\tilde{\mu }$ aimed at completing the instance of Equation (2).

The model selection procedure is based on a well-defined internal cross validation scheme. The original dataset is initially splitted in training and test datasets. The two datasets are then normalized to zero mean. The training set is further randomly partitioned in *s* subsamples $X_{1},....,X_{s}$, with *s* depending on the training set cardinality. The method stage I, for each subset $X_{i}$, builds a classifier using the remaining s-1 subsets as training set, and then validates it on $X_{i}$. The validation error is evaluated as the average error over the *s* subsets for each parameter *τ*. The optimal parameter $\tau_{opt}$ is selected as the one minimizing the validation error.

The stage II of the method builds a family of classifiers on the entire training set with $\tilde{\tau }=\tau_{opt}$ and for increasing values of *μ*. Each classifier returns a test error and a list of selected features, indexed with the relative value of *μ*.

#### 2.4.2. Support Vector Machines

Support Vector Machines (SVM) [63,64] are an effective approach for pattern classification and feature selection. Basically, SVM belong to the category of kernel methods, which generate non-linear decision boundaries among classes of patterns through methods designed for linear classifiers. Because of their good performances and readiness to use, SVM have been used in several application contexts like, for example, hand writing recognition, robotics, object recognition, intelligent vehicles field for scene understanding and 3D model classification [65,66,67].

In the field of marine sciences, SVM are used into a wide range of application contexts. SVM are used for classifying micro-zooplankton [2,3], they are used into a computer vision system aimed at continuously monitoring the fish eating activity [68] and are also used for detecting and classifying coral reef organisms [69,70].

According to reference [64], a classifier based on SVM is stated as the problem defined in

(3) $$\begin{array}{c} \min_{w,b,\xi }\left\{\frac{1}{2}w^{⊺}w+C\sum_{i=1}^{l}\xi_{i}\right\}\\ \mathrm{subject}\;\mathrm{to}:\;y_{i}\left(w^{⊺}\phi \left(x_{i}\right)+b\right)\geq 1-\xi_{i} \end{array}$$

where $\xi_{i}$ are the slack variables used to control the overfitting on the training set and *C* controls the balance between training accuracy and the margin width between positive and negative examples.

To solve the problem defined in Equation (3) several kernel functions have been defined, as discussed in reference [65]. The Gaussian kernel defined in Equation (4) has been used in the experiments proposed in Section 3.

(4) $$\kappa \left(x_{i},x_{j}\right)=\exp(\gamma ||x_{i}-x_{j}{\left|\right|}_{2}^{2}).$$

Beside the classification task, SVM can also perform feature selection through the Recursive Feature Elimination (RFE) algorithm [64,71]. RFE iteratively removes features corresponding to the SVM weight vector components that are smallest in absolute value; these features provide a lower contribution to the classification and are therefore removed [71]. The RFE method operates three steps: (i) training the classifier; (ii) computing the ranking criterion for all features; (iii) removing the features with smallest ranking criterion.

For identifying the subset of image-features maximizing the recognition performance, the RFE is nested within a K-fold CV framework, where the RFE is run iteratively for each CV training set. The first RFE iteration removes the feature with the smallest rank; at the second iteration the two features with the smallest ranks are removed and so on until just one feature is maintained and all the other features are removed. For each RFE iteration the selected features are used for training a new SVM based on the Gaussian kernel, where the parameters *C* and *γ* are chosen through a grid-search strategy. The learnt SVM is then validated on the CV validation set. The best performing SVM is then recorded together with the corresponding selected image-features and the *C* and *γ* values. After the whole CV procedure, the image-features can be ranked according to the number of times they were selected by the RFE.

#### 2.4.3. Genetic Programming

Genetic Programming (GP) is an evolutionary computation methodology that learns how to accomplish a given task [41,72,73]. GP generates the solutions to the given task starting from an initial population of mathematical expressions, that are randomly generated on the basis of a set of mathematical primitives, constants and variables. The initial solutions are improved by mimicking the selection processes that naturally occur in biological systems through the Selection, Crossover (i.e., the genetic operation that mixes the information contained in the two parent individuals into an offspring individual) and Mutation genetic operators [41]. In the proposed work, the binary classifiers evolved by the GP approach are expressed as mathematical functions, whose variables correspond to the image-features discussed in Section 2.3.4.

One of the most important characteristic of the GP approach is that no a-priori assumption is needed on the mathematical form of the evolved classifiers. Indeed the contribution of the image-features to the definition of the binary classifiers is not necessarily a simple linear combination. Even if strong non-linear primitives can be used, as shown in reference [74], the mathematical primitives used in this work are capable to capture different degrees of non-linearity ranging from simple classifiers based on the addition, subtraction, multiplication and division to more complex expressions based on square-root, logarithm and several trigonometric primitives.

The initial population is created with randomly computed binary classifiers and each generated classifier *c* is evaluated on the set of examples *E* defined in Equation (1) through the fitness function defined as

(5) $$\begin{array}{c} F\left(c\right)=\frac{1}{\left|E\right|}\sum_{(x,y)\in E}\left|J_{C}\left(x\right)-y\right|,\\ J_{c}\left(x\right)=\left\{\begin{array}{cc} 1 & \mathrm{if}\;eval(c(x))>0\\ 0 & \mathrm{otherwise} \end{array}\right., \end{array}$$

where eval(C(x)) returns a real number obtained by instantiating the variables of the classifier *C* with the image-features corresponding to the examples (x,y)∈E. The value of F(C)=0 corresponds to the best fitting classifier that produces zero classification errors during the training phase.

The classifiers which better fit the examples in *E* have higher probability of generating the new classifiers, which are the next generation of functions. In the proposed experiments, the formation of new populations of classifiers stops when a specified number of generations is reached. The following steps summarize the GP procedure used in this work to evolve the binary classifiers:

1. Randomly generate the initial parent population based on a set of mathematical primitives, constants and variables;
2. Evaluate the fitness of each individual;
3. Select two parent individuals for reproduction, according to their fitness: the individuals with higher fitness have greater probability to mate;
4. Determine whether to apply the crossover to the two parents to reproduce offspring, or whether to clone one parent to the next generation; determine whether mutation occurs on the offspring individual;
5. Repeat the steps 3 and 4 until the predetermined population size is reached;
6. Use the offspring population as a new generation and return to step 2. This is iterated until the stop criterion is met.

The more the GP procedure iterates through the steps 1 to 6, the higher is the probability that some evolved classifiers better classify the set of examples *E*. Nevertheless, by increasing the number of iterations, the probability that the evolved classifiers over-fit the set of examples *E* increases.

According to the methodology proposed in reference [75], the best classifier of the final generation is selected and the whole procedure is repeated within a K-fold CV framework. The output of the K-fold CV is a population-pool of classifiers that is further analysed for selecting the most relevant image features and for identifying the most effective binary classifiers.

An image-feature is deemed as relevant if it appears in the population-pool more often than by chance. A statistic test is used to identify the relevant image-features. In this test the null-hypothesis claims that all the image-features have the same probability to appear in the classifiers of the population-pool, while the alternative hypothesis claims that at least one image-feature occurs more often than the other image-features.

The problem of identifying whether an image-feature is relevant or not, is brought back to a Bernoulli trial defined on the classifiers of the population-pool that involve that feature.

According to the proposed statistic test, the probability to make a mistake by rejecting the null-hypothesis is represented by the p-value based on the Bernoulli trial. In the experiments proposed in Section 3.3, the *p*-value has been selected to be equal to 0.001, as suggested in reference [76]. The selected *p*-value corresponds to a specific number of image-feature occurrences th. The image-features whose occurrence is larger than th are deemed as relevant.

Some classifiers in the population-pool are based only on relevant image-features, other classifiers are based on both relevant and not relevant image-features. Only the former classifiers should be considered reliable for recognizing the jelly specimens, while the latter are based on image-feature that provide a random contribution to the classifier and thus are considered not capable to generalize the dataset *E*.

The population pool contains many individuals, that can be used to build a binary classifier used to recognize unknown RoIs. The strategy experimented in this work for building such a classifier is an ensemble E of individuals as defined in Equation (6):

(6) $$\begin{array}{c} E\left(r\right)=\left\{\begin{array}{cc} 1 & \mathrm{if}\;\sum_{c\in C_{ens}}\bar{J_{c}\left(r\right)}>0\\ 0 & \mathrm{otherwise} \end{array}\right.,\\ \bar{J_{c}\left(x\right)}=\left\{\begin{array}{cc} 1 & \mathrm{if}\;eval(c(r))>0\\ -1 & \mathrm{otherwise} \end{array}\right., \end{array}$$

where *r* is the unknown RoI to be classified, $C_{ens}$ is the subset of individuals of the population pool used in the ensemble and eval(c(r)) is the real number obtained by evaluating the classifier *c* as defined in Equation (5).

## 3. Results

The dataset defined in Equation (1) and used for the proposed experiments is the ground-truth set described in Section 2.2. It consists of 545 examples labelled as positive, i.e., RoIs containing jelly specimens, and negative, i.e., RoIs containing only water, suspended particulate, litter, algae and fishes. Each RoI is characterized by the 11 image-features discussed in Section 2.3.4.

According to the training and validation procedure described in Section 2.4, each example of the ground truth has been labelled within two classes: 1 if the RoI contains a jelly specimen, 0 otherwise. The ground truth consists of 211 positive examples (tagged with 1) and 334 negative examples (tagged with 0). In order to exhaustively exploit the available dataset and, in the meanwhile, to keep it balanced, the working dataset has been composed by keeping all the positive examples and by randomly selecting 211 out of 334 negative examples.

The three pattern recognition methods discussed in this paper have been run within a 10-fold stratified CV framework [57], where for each fold the 75% of the examples is further randomly subsampled 10 times for both training and validation sets. This CV approach has been adopted in order to enforce the generality of the obtained results.

This procedure yields to have one hundred runs of each experimented recognition method. At the end of the runs, the recognition performances of the methods have been evaluated by computing the average and standard deviation of Accuracy (ACC), True Positive Rate (TPR), False Positive Rate (FPR) and False Negative Rate (FNR) defined as

(7) $$\begin{array}{c} ACC=\frac{TP+TN}{TP+FP+FN+TN}\\ TPR=\frac{TP}{TP+FN}\\ FPR=\frac{FP}{FP+TN}\\ FNR=\frac{FN}{FN+TP} \end{array}$$

where TP, FP, TN and FN represent True Positive, False Positive, True Negative and False Negative recognitions respectively.

All the CV runs have been also used for tuning the specific parameters involved by the three recognition methods and to estimate the reliability of the relevant features identified by the three methods.

### 3.1. Elastic Net Based on Tikhonov Regularization

The Elastic Net based on Tikhonov Regularization method implementation used for the presented experiments has been realized using the L1L2Py Python package [77], which performs feature selection by means of $l_{1}l_{2}$ regularization with double optimization.

The implemented algorithm operates the two stages described in Section 2.4.1. According to the Equation (2), the stage I aims at selecting the optimal sparsity parameter $\tau_{opt}$ within the internal cross validation loop for a fixed and small value of the regularization parameter *μ*. The parameter *τ* mainly controls the sparsity of the model: the bigger it is, the more sparse and less accurate is the model and viceversa.

The algorithm first evaluates $\tau_{max}$, that is the value of *τ* for which Equation (2) has a void solution. All solutions with *τ* < $\tau_{max}$ have at least one non-null coefficient in the model vector *β*. In this stage the algorithm finds the $\tau_{opt}$ value in the *τ* range, for which the model *β* is not void, minimizing the prediction error via the internal CV scheme.

For the value of $\tau_{opt}$, as estimated in stage I, the stage II identifies the set of relevant lists of features for increasing values of the correlation parameter *μ*. As suggested in references [40,58], *μ* range is set as a geometric progression and it is defined as $\mu \in [10^{-10},10^{1}]$ with a multiplying step equal to 10.

Since the method is executed within the CV framework described above and resulting in one hundred runs, at the end of the procedure one hundred models, i.e., one hundred $\tau_{opt}$, were obtained. For each $\tau_{opt}$, the lists of relevant variables have same prediction power. The less sparse but more regularized solution (minimum value of *τ* and maximum value of *μ*) was selected in the set of (τ,μ) pairs.

As the focus is set on the feature selection capability of the method, ten different ranges for *τ* were defined with decreasing value of $\tau_{min}$, in order to analyze the performances of different models characterized by decreasing sparsity. So *τ* was set ranging as $\tau \in [\tau_{min}=\tau_{max}\cdot 10^{-t},\tau_{max}\cdot 10^{-t}+1]$ where t∈[1,10]. For each value of *t*, the method was run in the CV framework, thus generating ten sets of one hundred models to be ranked on the basis of the performance indicators.

**Figure 3:**
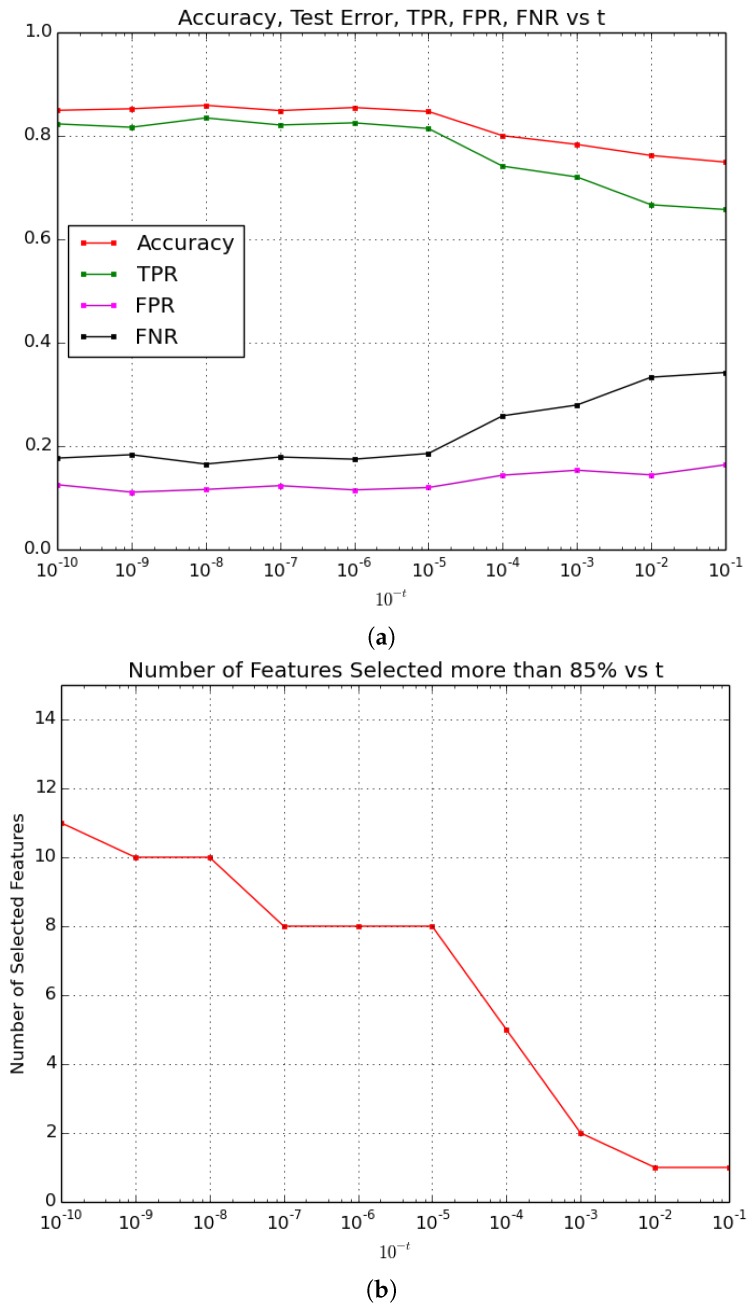
Elastic Net based on Tikhonov Regularization performance indicators (**a**) and number of features selected more than 85% of times (**b**) for different values of *t*. $10^{-t}$ is the parameter controlling the interval where *τ* ranges, as $\tau \in [\tau_{min}=\tau_{max}\cdot 10^{-t},\tau_{max}\cdot 10^{-t}+1]$. The bigger is *t*, the smaller are the values assumed by *τ*, the less sparse is the selected model.

Figure 3a shows the behaviour of Accuracy, True Positive Rate, False Positive Rate and False Negative Rate for each value of *t*, while Figure 3b presents the number of features selected more than 85% of times for each value of *t*. Results highlight different performances of the prediction Accuracy connected with the number of selected features. From the evolution of the indicator curves depicted in Figure 3a, it turns out that the performances of the method are equivalent with *t* ranging in [5,10], as with these values of *t* the Accuracy remains stable in the interval (0.847, 0.859), the True Positive Rate is stable in the interval (0.814, 0.835), the False Positive Rate is stable in the interval (0.111, 0.116) and the False Negative Rate is stable in the interval (0.165, 0.183).

As expected, Figure 3b shows that the number of the selected features is higher in the *t* interval where the model is more accurate and it is lower where the model is less accurate but more sparse. In particular, for *t* assuming values in [5, 10], 8 to 11 features are selected by the method more than 85% of the times. For *t* assuming values in [5, 7] only 8 features are selected, and the highest Accuracy is obtained for *t* = 6.

Table 3 summarizes the performance of the method for *t* in [1, 4] and in [5, 10] (average of the indicators in the intervals are reported), and highlights the values of the performance indicators for t=6.

According to this fact, *t* = 6 minimizes the computational cost of the recognition task and maximizes its accuracy, so it is the value chosen for the comparison aimed at selecting the method to be run onboard the GUARD1 system.

### 3.2. Support Vector Machines

According to the approach described in Section 2.4.2, the training of the RFE-SVM is nested within the K-fold Cross Validation approach described at the beginning of Section 3, with K=10.

For each sub-sampling, SVM based on a linear kernel [71] are iteratively trained for performing the RFE, as described in Section 2.4.2. For each iteration of the RFE, the selected features are used for training the SVM based on the Gaussian kernel where the parameters *C* and *γ* were obtained through a grid search in the range [0.1,100] and in the range [10,10000] respectively.

The obtained SVM were then evaluated in the corresponding CV sub-sampled validation set and, for each CV sub-sampled fold, the best performing SVM were recorded. In this way at the end of the whole CV framework, 100 SVM were generated, each of them based on a specific set of image-features selected through the RFE approach.

**Figure 4:**
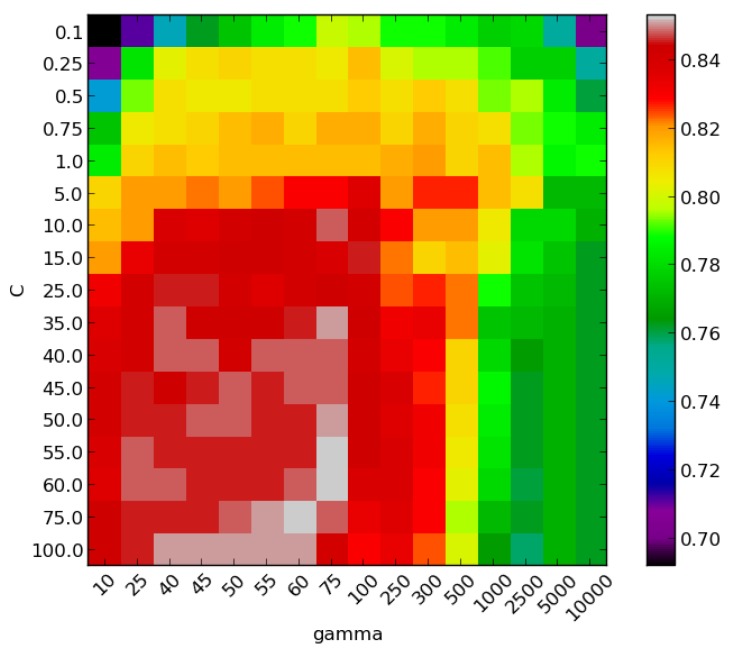
Accuracy values corresponding to the *C* and *γ* grid search, for the selected Support Vector Machines (SVM) classifier.

The occurrences of each image-feature were then summed in order to verify their persistence. Within the 100 classifiers obtained through the CV scheme, the one maximizing the Accuracy and minimising the number of used features is chosen for the comparison aimed at selecting the method to be run onboard the GUARD1 system. Figure 4 shows the accuracy values corresponding to the *C* and *γ* grid search, for the selected classifier. This classifier utilizes C=55, γ=75 and uses 8 features.

The RFE-SVM used in the described experiments were implemented using the Python scikit-learn package [78].

### 3.3. Genetic Programming

In order to evolve the GP-based classifiers described in Section 2.4.3, several parameters have to be chosen. For example the set of mathematical primitives the individuals are based on, the number of individuals of the initial population, the number of generations the individuals evolve through and the specific parameters driving the crossover and the mutation among individuals, as explained in reference [72] and summarized in Table 4.

After several experiments, the GP parameters have been selected in order to reduce the overfitting effects on the training and validation sets and to increase the generalization capability of the evolved classifiers.

The variables used for the generation of the initial population are the image-features described in Table 2, and the constants correspond to *k* real numbers randomly selected in the range [-10,10], where *k* is a natural number randomly selected in the range [0,10]. The initial population consists of 1000 individuals and it is evolved, at most, for 500 generations. Each individual is a mathematical expression represented as a rooted tree whose maximum depth is 4. Greater values of depth could be used with the risk of producing bloated mathematical expressions susceptible to over-fitting. The whole population is generated according to the ramped half-and-half technique [41,72]. The raw fitness of an individual is defined by the Equation (5), and the scaled fitness represents how the individual fits relatively to the current population.

The individuals are selected for reproduction depending on their fitness, according to the roulette wheel strategy: the probability that an individual is selected is proportional to its scaled fitness value. In the proposed experiments, the probability that a crossover happens between two selected individuals is 0.9. If crossover does not happens only one individual is selected and cloned to the next generation. After the crossover or cloning, the mutation occurs with probability $2\times 10^{-4}$. Although the crossover is the most important genetic operation used for evolving effective classifiers, a crossover rate smaller than 1 guarantees the continuity of the best individuals in successive generations. The mutation rate is set to a small value in order to reduce the introduction of random components during the evolution of the classifiers.

Besides the selection and mating, at each generation, the best individual is cloned to the next generation (elitism) to obtain better classifiers. Finally the GP procedure ends when the maximum number of generations is attained or when an evolved individual has raw fitness equal to 0, corresponding to zero classification errors during the training phase as defined in Equation (5).

**Figure 5:**
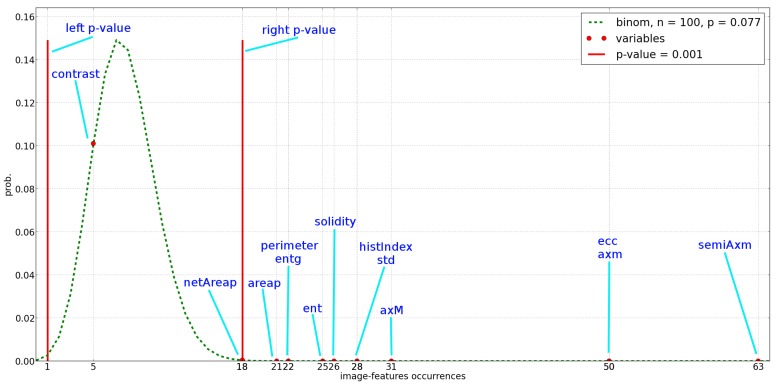
Relevance of the image-feature, according to the test statistics discussed in Section 2.4.3. The abscissa represents the occurrences of the image-features within the population pool. The ordinate represents the probability an image-feature occurred in the population pool is selected. The two red vertical lines represent the two-tails *p*-values.

According to the feature selection method proposed in Section 2.4.3, the relevant image-features are identified by analysing their occurrences among the classifiers of the population pool. Figure 5 shows the probability distribution of the image-feature occurrences (green dotted line) according to the Bernoulli trial. The red filled circles represent the number of occurrences of the image-features in the population pool, while the vertical red lines represent the two-tails *p*-value equal to 0.001 used to select the image-features. Actually the image-features on the right of the right vertical line are deemed as relevant.

**Figure 6:**
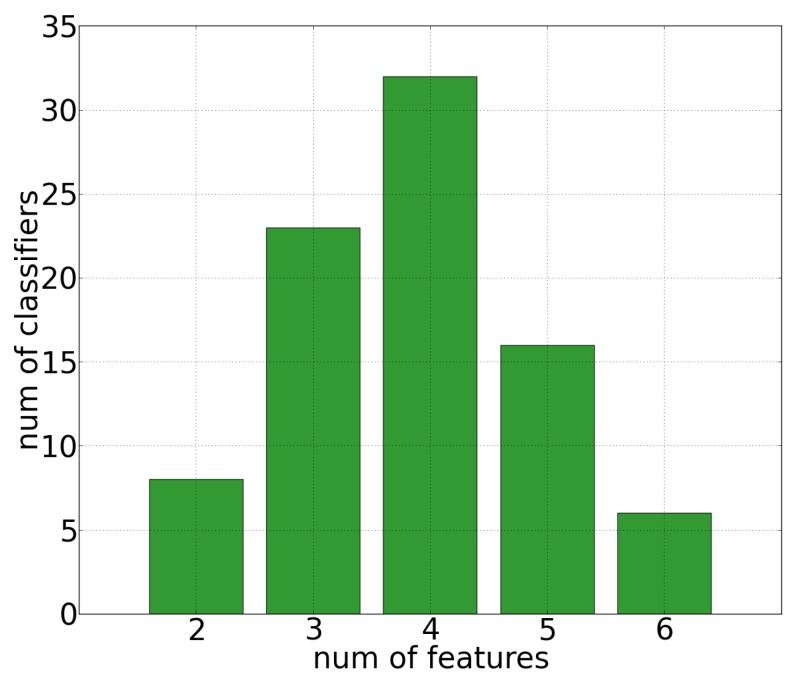
Number of population pool classifiers generated by GP (y-axis) with respect to the number of involved variables (x-axis).

As shown in Figure 5 all the image-feature can be considered relevant but “contrast”. Nevertheless, the image classifiers of the population pool are based only on small sub sets of relevant image-features, varying from a minimum of 2 to a maximum of 6 image-features as shown in Figure 6.

Since this work is aimed at identifying the simplest binary classifier to run on the GUARD1 imaging device, the ensemble defined in Equation (6) has been built by considering only the individuals of the population pool involving the smallest number of image-features. The ensemble proposed in this work is based on the most relevant variables shown in Figure 5, that is *semiAxm*, *axm* and *ecc*. According to the generated population pool, only three binary classifiers involve only the previous relevant variables. These classifiers are shown in Table 5.

The occurrence percentage resulting form the GP based analysis is presented in Table 6 (third column), while Table 7 (third row) summarizes the average performance of the whole population pool.

The GP based approach used in the described experiments were implemented using the Python Pyevolve package [79].

## 4. Discussion

As shown in in Table 7, there are no significant differences in the performances of the three methods in terms of prediction accuracy and other performance indicators. In fact their values lie within the standard deviation intervals of each method, making the differences among the values not significant in terms of recognition performances.

On the contrary, differences are highly evident with respect to the selection of the relevant features, as shown in Table 6, where the red values represent the relevant image-features selected by each method in its best run. Section 3.1 and Section 3.2 demontrate that TR and SVM achieve their best trade-off between recognition performance and number of selected features by using 8 image-features.

Also Section 3.3 states that the classifiers evolved by GP involve a smaller number of image-features, in fact, the winner ensemble of classifiers utilizes just 3 image-features.

Concerning the feature selection capability of the three methods, it has to be noticed that TR is a forward selection method that starts from an initial minimal set of relevant features (the most sparse possible model) and then produces models of increasing cardinality by exploiting the correlation patterns linking the features in cooperating groups.

On the other hand, the RFE based on SVM is a backward elimination method that discards features not deemed as relevant during the construction of the classifiers.

Also the proposed GP based approach should be considered a backward elimination method where at the beginning of the selection process all the image-features are hypothesized to be relevant and the statistic test defined in Section 2.4.3 removes all the not relevant image-features.

In this study the GP method proved to select the minimum number of features with respect to the other methods, while achieving the same performances. On the basis of this result, the GP technique has been selected to be the one to be run operationally onboard the GUARD1 system, because it is the method maximing the recognition performances while minimizing the computational load, due to its selecting the minimum number of image-features in the classification task.

### The Operational Test

The comparison among the recognition methods resulted in the choice of the GP algorithm as it is the more efficient in terms of trade-off between recognition performances and selected image-features. Thus, the GP based tool has been installed onboard the GUARD1 system to be operationally run during the system missions.

In order to perform an operational test of the whole system, the GP classifier was run on the entire dataset collected during the survey in Baia Santa Teresa described in Section 2.2. The 9282 images were annotated by the team of marine biologists and experts of gelatinous zooplankton and build the ground-truth dataset that has been used to quantitatively evaluate the recognition performances of the GUARD1 system running the operational recognition algorithm, i.e., GP.

**Figure 7:**
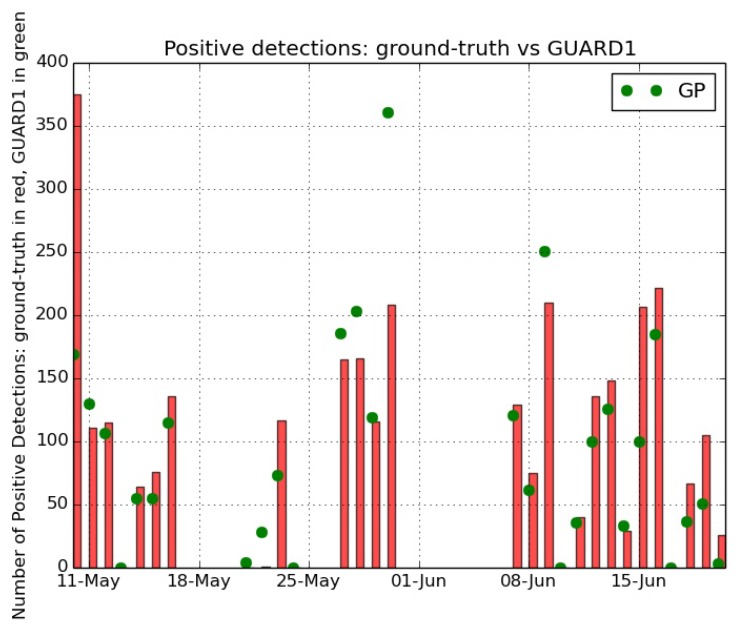
The recognition results of the GP method applied on the whole image time series collected in the Ligurian Sea during the bloom of gelatinous zooplankton in the period May–June 2013. The time series has been annotated by experts and builds a ground-truth dataset. The vertical red bars represent the number of jellies identified by the biologists in the corresponding day, and the green markers represent the number of jelly specimens recognized by the GP method. The Pearson correlation evaluated in time between the number of identified specimens from the ground-truth and the number of positive detections by the GP algorithm is 0.79.

Figure 7 shows the results of the operational test, by comparing the positive detections of the GP algorithm versus the jelly identifications of the ground-truth dataset. The test results highlight the capability of the method in identifying jelly specimens, as the positive detections by the GP algorithm capture in a satisfactory way the trend during time of the presence of jellies in the monitored area. This agreement is strongly visible along the overall time series, even if two critical time intervals are present. These criticalities refer to the period 21–24 May 2013 and to 30 May 2013.

**Figure 8:**
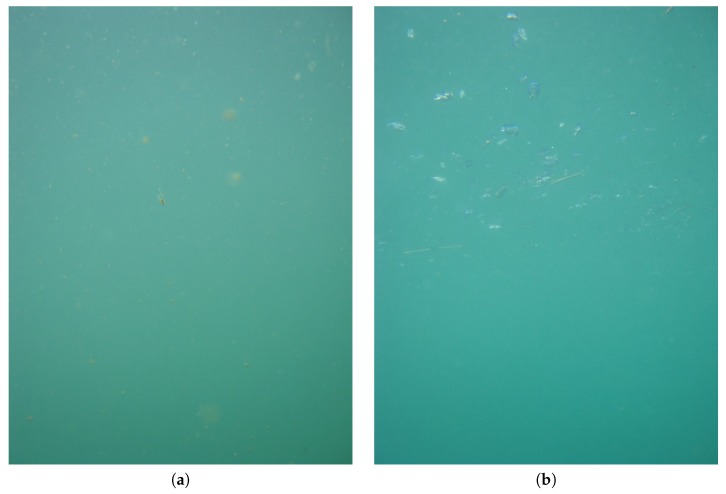
Examples of images including massive presence of particulate (**a**) and of the hydrozoan *Velella velella*, visible in the upper part of (**b**), shaded in blue.

In the days 21–24 May a massive presence of suspended particulate has been recorded in the images, as shown in Figure 8a. This fact influenced the recognition by pushing the presence of many False Positives caused to the mis-identification of particulate with jellies.

On 30 May 2013 a bloom of the hydrozoan *Velella velella* occurred, as captured by the collected images (Figure 8b shows an example of an image acquired in that day). This organism has been identified by the GP method as a ctenophore, thus pushing the high number of positive detections, but it has not been annotated by the biologists since it is not a target of the research. These facts caused the differences between ground-truth identifications and GP recognitions.

Anyway, the strong similarity in recognition trends proves how the GP algorithm captures the temporal dynamics of the abundance of the target species and confirms the robustness of the method for the jelly recognition. This statement is supported by the high value of the Pearson correlation evaluated in time between the number of identified specimens from the ground-truth and the number of positive detections by the GP algorithm: 0.79.

**Figure 9:**
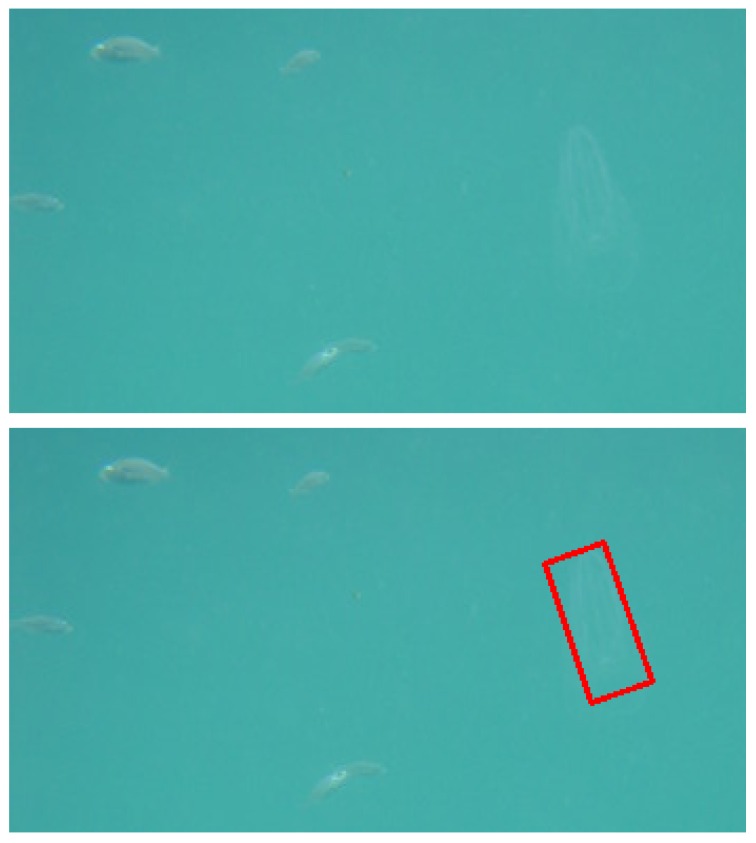
Detail of an image acquired by the GUARD1 system (top panel). A very transparent specimen of Ctenophore is visible on the right side of the panel, close to a small school of fish (on the left side of the panel). In the lower panel the same detail is presented, where the Ctenophore is automatically detected by the recognition algorithm: it is highlighted with the red bounding box and discriminated from the fishes.

The top frame of Figure 9 shows a detail of an acquired image, where a very transparent specimen of ctenophore (right side) is close to a small school of fishes (left side). The framed scene shows the typical image content of the water column application context, where the background is just water and the foreground consists of floating subjects, like for example fishes, algae, marine litter and jellies. The lower panel of Figure 9 shows the same detail where the ctenophore is automatically detected by the recognition algorithm, highlighted with a red box and discriminated from the fishes.

**Figure 10:**
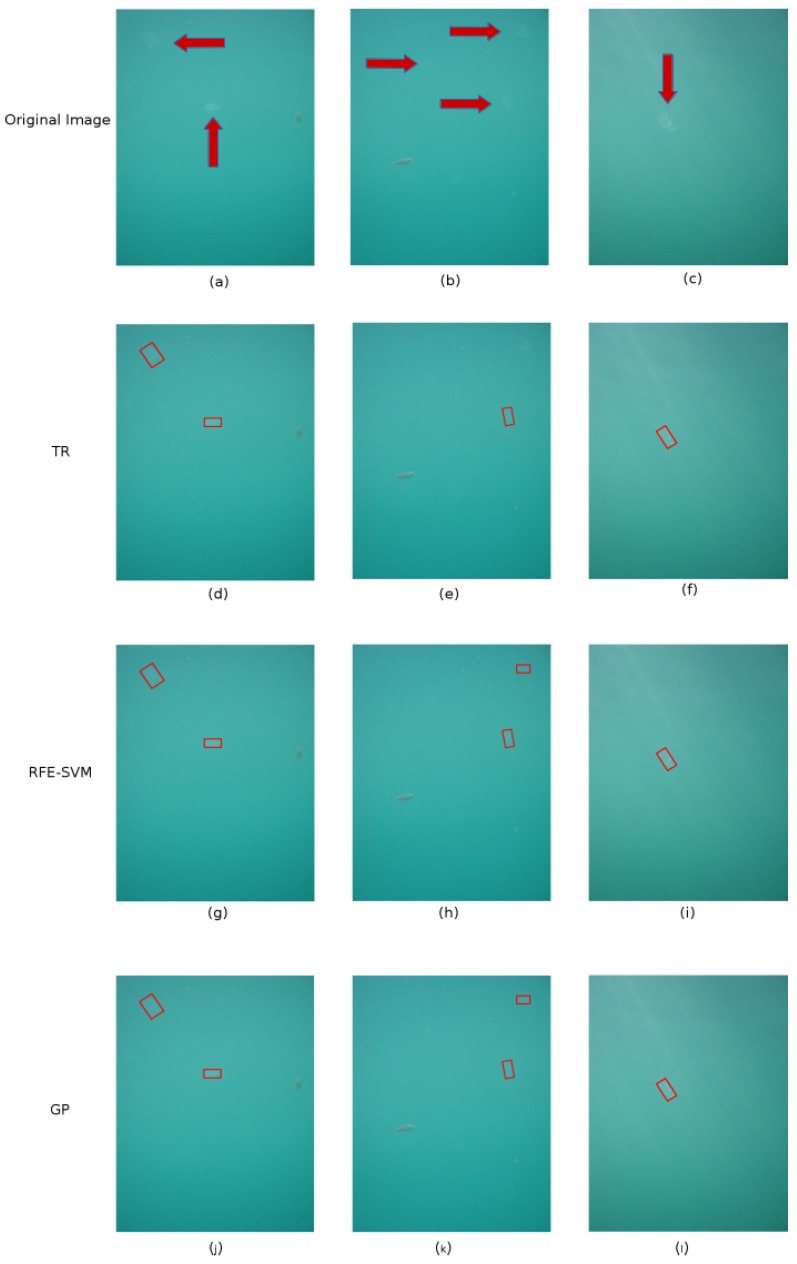
Some examples of the image time series collected during the gelatinuous zooplankton bloom in the Ligurian Sea and the corresponding recognition results performed by the three methods. The first row of figure show three examples of images acquired by the GUARD1 imaging system (**a**–**c**), where the jelly specimens are highlighted by red arrows. The last three rows present the recognition results of the three methods: TR on the second row (**d**–**f**); RFE-SVM on the third row (**g**–**i**); and GP on the fourth row (**j**–**l**).

Figure 10 shows some examples of the image time series and the corresponding recognition results performed by the three methods.

The first row of the figure shows three images acquired by the GUARD1 imaging system, where the jelly specimens are highlighted by red arrows. In Figure 10a two specimens of ctenophora are framed close to a floating organic material (probably algae); in Figure 10b a fish is close to three ctenophores and in Figure 10c some light reflections run over a specimen of ctenophore. Panels from d to n correspond to recognition results obtained by the three methods discussed in Section 2.4. The figure shows how the three methods perform in a very similar way in recognizing the jelly specimens and avoiding other floating objects.

## 5. Conclusions

The GUARD1 autonomous imaging system is described and its potential to monitor and automatically recognize gelatinous macro-zooplankton (from 1 mm to 100 cm in size) in the ocean interior at various scales in space and time is demonstrated.

Focus has been put on the problems and the proposed solutions for the onboard automatic recognition of macro gelatinous zooplankton. Three different recognition methodologies, namely Tikhonov Regularization (TR), Support Vector Machines (SVM) and Genetic Programming (GP) have been implemented and their performances have been compared in terms of recognition performances and computational cost, since a key requirement for algorithms to be run onboard an autonomous system designed for long lasting deployments is the minimization of the system energy consumption.

The proposed methodologies include feature selection schemes for identifying the most suitable image features capable to discriminate gelatinous zooplankton from other floating objects captured by the images (e.g., fishes, litter, algae, light reflections), in order to optimize the recognition performances and the computational cost.

The aim of the performance comparison was also the evaluation of the recognition capability of two novel (for underwater classification applications) recognition methods (i.e., TR and GP) with SVM, which is a more consolidated methodology for underwater recognition and classification.

The performances of the methods have been evaluated within a well-defined CV framework based on a ground-truth dataset of annotated images of gelatinous macro-zooplankton acquired by the GUARD1 system in the Ligurian Sea in the period May–June 2013.

The overall results indicate that the three methods provide high accuracy in the recognition of gelatinous zooplankton, showing a good capability in robustly selecting relevant features, thus avoiding computationally consuming preprocessing stages, as required for running on an autonomous imaging system designed for long lasting deployments, like the GUARD1.

In particular, the three methods do not show significant performance differences in terms of prediction accuracy and performance indicators, since the achieved Accuracies range in (0.847, 0.856), the True Positive Rates range in (0.825, 0.846), the False Positive Rates range in (0.115, 0.149) and the False Negative Rates range in (0.154, 0.175). The comparison proved that the three proposed methods provide good generalization capability.

On the contrary, differences are evident in terms of selection of the relevant features. TR and SVM methods achieve their best performances by selecting 8 out of the available 11 features, while GP technique selects 3 features out of 11 to classify the dataset, achieving the same performances of the other two methods.

Hence the GP algorithm turns out to be the most efficient in avoiding computationally consuming preprocessing stages and it has been selected to be run operationally onboard the GUARD1 system, because it is the method maximing the recognition performances while minimizing the computational load in the classification task.

An operational assessment of the whole system has been performed by running the GP classifier on the entire dataset collected in the Ligurian Sea in the period May-June 2013. The dataset, consisting of 14,522 RoIs extracted from 9282 images, has been previously annotated by a team of marine biologists and gelatinous zooplankton experts in order to establish a ground-truth for the quantitative evaluation of the recognition performances.

The test results highlighted the capability of the GP algorithm in capturing the temporal dynamics of the abundance of the target species and confirmed the robustness of the GP method for the jelly recognition. In fact the temporal trend of the positive detections by the classifier is very similar to the one identified by the ground-truth, as confirmed by the high value of the Pearson correlation evaluated in time between the number of identified specimens from the ground-truth and the number of positive detections by the GP algorithm, i.e., 0.79.

The GUARD1 system is thus ready to operate missions for autonomous monitoring of gelatinous zooplankton abundance in the open and interior ocean.

Furthermore, thanks to the very general character of its hardware and software architectures, several applications related to recognition, counting and classification of other zooplankton taxa, fishes, or marine litter can be envisioned for GUARD1.

A multidisciplinary approach for studying the connectivity among Marine Protected Areas (MPAs) is proposed [80]. In particular, the connectivity between MPAs can be investigated by combining direct observations of organisms provided by GUARD1 with surface water current measurements, integrated by Lagrangian variational analysis models of dispersion.

In the framework of regional experiments aimed at studying gelatinous zooplankton dynamics, e.g., horizontal and vertical distribution of jellies and their mechanisms of vertical migration or transport by currents, the GUARD1 system can operate hosted onboard autonomous mobile platforms, including drifters for surface information, or profiling floats or gliders to investigate vertical migration and distribution.

Once the communication module is implemented, the GUARD1 system can also be applied in continuous patrolling activities aimed at detecting the presence of gelatinous zooplankton both in open ocean and in coastal seas, for providing early warnings in case of jelly invasion. For these applications the system can be hosted by remotely operated platforms, such as gliders for long term deployments, or powered AUVs for specific, short time deployments.

The framework of global monitoring for recovering large scale and long-term information is for sure a more challenging scenario for the application of the GUARD1 system. It could operate onboard gliders, expendable drifters or Argo floats. For this kind of activities, the recognition algorithm could be easily integrated in order to allow the system to not only identify known species, but also to detect and communicate image regions whose content can be relevant, even if unknown.

The future developments of the system will be aimed at these improvements.

## Figures and Tables

**Table 1:** Summary of the main imaging devices developed for automatic monitoring of zooplankton. For comparison purposes, the functionalities of the GUARD1 system, object of the present paper, are reported.

Imaging System	Hosting Platform	Monitoring Target	System Functionalities
LAPIS [23]	Towed by vessel	Gelatinous zooplankton (size (1 cm–100 cm))	Imaging
			No recognition
			No classification
ZOOVIS [24]	Towed by vessel	Gelatinous zooplankton (size < 5 mm)	Imaging
			Offline recognition and classification [25]
ISIT [26]	Towed by vessel	Bioluminescent events produced by deep zooplankton	Imaging
			Offline automatic recognition
ICCD [27]	Fixed and mobile	Bioluminescent events produced by deep zooplankton	Imaging
			Offline automatic recognition
VPR [28,29,30]	Towed by vessel ROV	Zooplankton (size (1 μm–10 cm))	Imaging
			Offline automatic recognition
			Offline automatic classification
			(Zooprocess [3,31])
UVP [32,33]	Towed by vessel ROV	Zooplankton (size (1 μm–10 cm))	Imaging
			Offline automatic recognition
			Offline automatic classification
			(Zooprocess [3,31])
ISIIS [34]	Towed by vessel	Zooplankton (size (1 μm–13 cm))	Imaging
			Offline automatic recognition
			Offline automatic classification
OPC [35]	AUV (Tested in lab)	Micro plankton (not jellies)	Imaging
			Onboard automatic particle counting
GUARD1 [36,37,38]	Fixed and mobile	Gelatinous zooplankton (size (1 mm–100 cm))	Imaging
			Onboard automatic recognition

**Table 2:** Features extracted from each single image Region of Interest (RoI), where *h* is the histogram of the RoI grey level pixel intensities.

	Feature Code	Feature Meaning	Feature Computation
**geometric**	*semiAxm*	minor semi-axis	
	*axm*	minor axis	
	*axM*	major axis	
	ecc	eccentricity	$\frac{\sqrt{axM^{2}-axm^{2}}}{axM}$
	*solidity*	solidity	$\frac{area(RoI)}{area(hull(RoI))}$
	*areap*	area expressed in pixels	
	*perimeter*	perimeter	
**texture**	*histIndex*	histogram shape index	$std\left(\frac{h}{\sum h}\right)$
	stdg	normalized grey	$\sqrt{\sum_{i=0}^{255}x{\left(i-\mu \right)}^{2}},$
		standard deviation	$\mu =\sum_{i=0}^{255}xi,\;x\in \left(\frac{h}{\sum h}\right)$
	*ent*	entropy	$-\sum x\log\left(x\right),\;x\in \left(\frac{h}{\sum h}\right)$
	*contrast*	normalized interior/ exterior contrast	$\frac{\left|mean(gInt)-mean(gExt)\right|}{mean(gInt)+mean(gExt)}$

**Table 3:** Summary of average performance indicators of Elastic Net based on Tikhonov Regularization for different intervals of the parameter *t*.

Indicator	t∈[1,4]	t∈[5,10]	t=6
ACC	0.765	0.852	0.855
TPR	0.682	0.822	0.825
FPR	0.154	0.117	0.115
FNR	0.318	0.178	0.175

**Table 4:** The Genetic Programming (GP) parameters used for the evolution of the binary classifiers for the jelly recognition task.

Mathematical Primitives	$\left\{+,-,*,{/}^{*},{\mathrm{sqrt}}^{*},\log^{*},\sin,\cos,\tan,\mathrm{atan}\right\}$
variables	the image-features summarized in Table 2
constants	*k* random numbers from the range [-10,10],
	where *k* is randomly selected in the range [0,10]
initial population	ramped half-and-half
individual max depth	4
population size	1000
max generations	500
raw fitness	defined in the Equation (5)
scaled fitness	linear scaling
selector method	roulette wheel
crossover rate	0.9
mutation rate	$2\times 10^{-4}$
elitism	true
termination criterion	max generations or raw fitness = 0.00

**Table 5:** The individuals of the population pool used to build the ensemble of binary classifiers defined in Equation (6).

$C_{1}=log\left(sAxm\right)+atan\left(ecc\right)-0.31+\sqrt{ecc}$
$C_{2}=log\left(atan\left(ecc\right)\right)+cos\left(ecc-sAxm\right)+\sqrt[{4}]{sAxm}+log\left(atan\left(sAxm\right)\right)$
$C_{3}=2*\sqrt{sAxm}+tan\left(log\left(ecc\right)\right)+log\left(sAxm\right)+cos\left(log\left(sAxm\right)\right)$

**Table 6:** Occurrence percentage of the features selected by the three methods. Values highlighted in red are the image-features used by the processing component of the GUARD1 imaging system.

	TR	RFE-SVM	GP
**sAxm**	**100**	**100**	**63**
**entg**	**97**	**100**	22
**stdg**	79	34	28
**axm**	**100**	**100**	50
**axM**	**100**	**100**	31
**ecc**	**100**	80	**50**
**sol**	**100**	**100**	26
**areap**	**100**	**100**	21
**per**	**100**	**100**	22
**hstI**	26	0	28
**ent**	81	**100**	25
**ctrs**	62	32	-

**Table 7:** Average and standard deviation (*in brackets*) of Accuracy (ACC), True Positive Rate (TPR), False Positive Rate (FPR) and False Negative Rate (FNR) the performance indicators for each recognition method.

PR Method	ACC	TPR	FPR	FNR
TR	0.855 (*0.055*)	0.825 (*0.069*)	0.115 (*0.081*)	0.175 (*0.069*)
SVM	0.847 (*0.061*)	0.844 (*0.084*)	0.149 (*0.090*)	0.155 (*0.084*)
GP	0.856 (*0.045*)	0.846 (*0.089*)	0.135 (*0.059*)	0.154 (*0.089*)
